# Triphenyl{(*E*)-4-[4-(phenyldiazenyl)phenyl]-4*H*-1,2,4-triazol-1-yl}boron

**DOI:** 10.1107/S1600536809036071

**Published:** 2009-09-09

**Authors:** Daisuke Urakami, Katsuya Inoue, Shinya Hayami

**Affiliations:** a Department of Chemistry, Graduate School of Science, Hiroshima University, 1-3-1 Kagamiyama, Higashi-Hiroshima 739-8526, Japan; b Institute for Advanced Materials Research, Hiroshima University, 1-3-1 Kagamiyama, Higashi-Hiroshima 739-8526, Japan; c Department of Chemistry, Graduate School of Science and Technology, Kumamoto University, 2-39-1 Kurokami, Kumamoto 860-8555, Japan

## Abstract

In the title compound, C_32_H_26_BN_5_ or [(C_14_H_11_N_5_)B(C_6_H_5_)_3_], the B atom is approximately tetra­hedrally coordinated. The diazo unit is in a *trans* conformation, which is generally more stable than a *cis* one for aromatic azo compounds. The crystal structure features very weak C—H⋯π inter­actions. The  dihedral angles between the central benzene ring and the terminal rings in the heterocycle are 62.64, 73.54 and 61.60°.

## Related literature

For the use of azobenzenes for holographic information storage, see: Rasmussen *et al.* (1999 ▶).
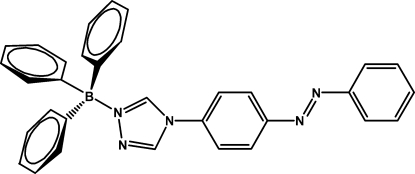


## Experimental

### 

#### Crystal data

C_32_H_26_BN_5_
*M* *_r_* = 491.39
Monoclinic, 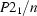

*a* = 17.4049 (6) Å
*b* = 7.1284 (2) Å
*c* = 21.0645 (7) Å
β = 97.0810 (15)°
*V* = 2593.52 (14) Å^3^
*Z* = 4
Mo *K*α radiation
μ = 0.08 mm^−1^
*T* = 113 K
0.40 × 0.20 × 0.10 mm

#### Data collection

Rigaku R-AXIS RAPID diffractometer
Absorption correction: multi-scan (Higashi, 2001 ▶) *T*_min_ = 0.971, *T*_max_ = 0.993
19971 measured reflections
5668 independent reflections
4068 reflections with *I* > 2σ(*I*)
*R*_int_ = 0.050

#### Refinement

*R*[*F* ^2^ > 2σ(*F* ^2^)] = 0.050
*wR*(*F* ^2^) = 0.138
*S* = 1.03
5668 reflections
343 parameters
H-atom parameters constrained
Δρ_max_ = 0.26 e Å^−3^
Δρ_min_ = −0.27 e Å^−3^

### 

Data collection: *PROCESS-AUTO* (Rigaku, 1998 ▶); cell refinement: *PROCESS-AUTO*; data reduction: *CrystalClear* (Rigaku/MSC, 2002 ▶); program(s) used to solve structure: *SHELXS97* (Sheldrick, 2008 ▶); program(s) used to refine structure: *SHELXL97* (Sheldrick, 2008 ▶); molecular graphics: Yadokari-XG (Wakita, 2000 ▶); software used to prepare material for publication: *SHELXL97*.

## Supplementary Material

Crystal structure: contains datablocks I, global. DOI: 10.1107/S1600536809036071/bx2228sup1.cif

Structure factors: contains datablocks I. DOI: 10.1107/S1600536809036071/bx2228Isup2.hkl

Additional supplementary materials:  crystallographic information; 3D view; checkCIF report

## Figures and Tables

**Table 1:** Hydrogen-bond geometry (Å, °)

*D*—H⋯*A*	*D*—H	H⋯*A*	*D*⋯*A*	*D*—H⋯*A*
C4—H3⋯*Cg*1^i^	0.95	2.91	3.822 (2)	162

Symmetry code: (i) 
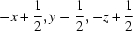
. *Cg*1 is the centroid of the C27–C31 ring.

**Fig. 1.**
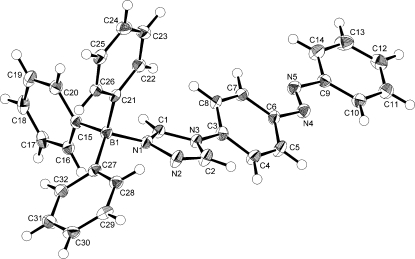
ORTEP drawing for the compound (I) showing 50% probability displacement ellipsoids.

**Fig. 2.**
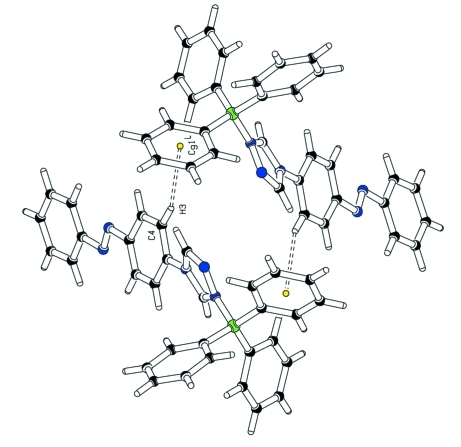
Packing structure for the compound (I).

## supplementary crystallographic information

### Comment

Photochromic materials are of interest as media for recording, storage, and reproduction of information in nonlinear optics and holography. Aromatic azo compounds occupy an important place among such materials, therefore many photoisomerizable azobenzene groups were synthesized to apply for photoswitchable systems or optical data storage device (Rasmussen *et al.*, 1999). Here we report the synthesis and crystal structure of the title complex. The addition compound of a triazole derivative with diazobenzene and a triphenylboron contains a B atom approximately tetrahedrally coordinated with a B—N distance of 1.636 (2) Å and B—C bond lengths averaging 1.625 (2) Å. The diazo moiety forms a *trans* conformation, and the bond distance N4—N5 is 1.2563 (19) Å. The crystal structure is stabilized by weak C—H···π (C27/C32) interactions between phenyl hydrogens from *(E)-4-(4-(phenyldiazenyl)phenyl-4H-1,2,4-triazole fragment* to phenyl rings from triphenylboron fragment. Fig 2, Table 1.

### Experimental

The compound (I) was synthesized by refluxed for an hour with (*E*)-4-(4-(phenyldiazenyl)phenyl)-4*H*-1,2,4-triazole (3 mmol, 0.748 g), sodium tetraphenylborate (4 mmol, 1.369 g) and iron chloride tetrahydrate (2 mmol, 0.390 g) in ethanol (50 ml). The resulting yellow solution was filtered and the single crystals of (I) suitable X-ray diffraction were obtained by slowly evaporation of ethanol.

### Refinement

All H atoms were positioned geometrically (C—H = 0.95 Å) and were refined as riding, with *U*_ĩso_(H) = 1.2*U*_eq_(C).

### Crystal data

**Table:**

C_32_H_26_BN_5_	*F*(000) = 1032
*M**_r_* = 491.39	*D*_x_ = 1.258 Mg m^−^^3^
Monoclinic, *P*2_1_/*n*	Mo *K*α radiation, λ = 0.71069 Å
Hall symbol: -P 2yn	Cell parameters from 13301 reflections
*a* = 17.4049 (6) Å	θ = 2.7–27.4°
*b* = 7.1284 (2) Å	µ = 0.08 mm^−^^1^
*c* = 21.0645 (7) Å	*T* = 113 K
β = 97.0810 (15)°	Block, orange
*V* = 2593.52 (14) Å^3^	0.40 × 0.20 × 0.10 mm
*Z* = 4	

### Data collection

**Table:**

Rigaku R-AXIS RAPID diffractometer	5668 independent reflections
Radiation source: fine-focus sealed tube	4068 reflections with *I* > 2σ(*I*)
graphite	*R*_int_ = 0.050
ω scans	θ_max_ = 27.4°, θ_min_ = 1.4°
Absorption correction: multi-scan (Higashi, 2001)	*h* = −21→22
*T*_min_ = 0.971, *T*_max_ = 0.993	*k* = −9→8
19971 measured reflections	*l* = −27→27

### Refinement

**Table:**

Refinement on *F*^2^	Primary atom site location: structure-invariant direct methods
Least-squares matrix: full	Secondary atom site location: difference Fourier map
*R*[*F*^2^ > 2σ(*F*^2^)] = 0.050	Hydrogen site location: inferred from neighbouring sites
*wR*(*F*^2^) = 0.138	H-atom parameters constrained
*S* = 1.03	*w* = 1/[σ^2^(*F*_o_^2^) + (0.0835*P*)^2^] where *P* = (*F*_o_^2^ + 2*F*_c_^2^)/3
5668 reflections	(Δ/σ)_max_ = 0.002
343 parameters	Δρ_max_ = 0.26 e Å^−^^3^
0 restraints	Δρ_min_ = −0.27 e Å^−^^3^

### Special details

**Table:**

Geometry. All e.s.d.'s (except the e.s.d. in the dihedral angle between two l.s. planes) are estimated using the full covariance matrix. The cell e.s.d.'s are taken into account individually in the estimation of e.s.d.'s in distances, angles and torsion angles; correlations between e.s.d.'s in cell parameters are only used when they are defined by crystal symmetry. An approximate (isotropic) treatment of cell e.s.d.'s is used for estimating e.s.d.'s involving l.s. planes.
Refinement. Refinement of *F*^2^ against ALL reflections. The weighted *R*-factor *wR* and goodness of fit *S* are based on *F*^2^, conventional *R*-factors *R* are based on *F*, with *F* set to zero for negative *F*^2^. The threshold expression of *F*^2^ > σ(*F*^2^) is used only for calculating *R*-factors(gt) *etc*. and is not relevant to the choice of reflections for refinement. *R*-factors based on *F*^2^ are statistically about twice as large as those based on *F*, and *R*- factors based on ALL data will be even larger.

### Fractional atomic coordinates and isotropic or equivalent isotropic displacement parameters (Å^2^)

**Table:**

	*x*	*y*	*z*	*U*_iso_*/*U*_eq_	
B1	0.21836 (11)	0.3083 (3)	0.04914 (9)	0.0190 (4)	
C1	0.29403 (9)	0.0119 (2)	0.10360 (8)	0.0197 (4)	
H1	0.2800	−0.0758	0.0702	0.024*	
C2	0.34594 (11)	0.1427 (2)	0.19029 (8)	0.0285 (4)	
H2	0.3765	0.1618	0.2304	0.034*	
C3	0.38777 (9)	−0.1882 (2)	0.17537 (8)	0.0201 (4)	
C4	0.41059 (10)	−0.2207 (2)	0.23988 (8)	0.0256 (4)	
H3	0.3942	−0.1399	0.2714	0.031*	
C5	0.45769 (11)	−0.3730 (2)	0.25743 (8)	0.0273 (4)	
H4	0.4750	−0.3949	0.3014	0.033*	
C6	0.48000 (9)	−0.4945 (2)	0.21125 (8)	0.0214 (4)	
C7	0.45478 (10)	−0.4616 (2)	0.14683 (8)	0.0219 (4)	
H5	0.4687	−0.5460	0.1153	0.026*	
C8	0.40960 (9)	−0.3068 (2)	0.12870 (8)	0.0217 (4)	
H6	0.3937	−0.2820	0.0847	0.026*	
C9	0.58832 (10)	−0.9199 (2)	0.21814 (8)	0.0225 (4)	
C10	0.62167 (10)	−0.9365 (3)	0.28142 (8)	0.0247 (4)	
H7	0.6102	−0.8473	0.3124	0.030*	
C11	0.67159 (10)	−1.0832 (3)	0.29885 (9)	0.0296 (4)	
H8	0.6944	−1.0950	0.3420	0.036*	
C12	0.68874 (11)	−1.2142 (3)	0.25356 (9)	0.0330 (5)	
H9	0.7238	−1.3137	0.2657	0.040*	
C13	0.65460 (12)	−1.1989 (3)	0.19090 (10)	0.0353 (5)	
H10	0.6659	−1.2888	0.1600	0.042*	
C14	0.60408 (11)	−1.0529 (3)	0.17306 (9)	0.0313 (4)	
H11	0.5802	−1.0435	0.1301	0.038*	
C15	0.17458 (9)	0.1567 (2)	−0.00001 (8)	0.0194 (4)	
C16	0.12558 (9)	0.0210 (2)	0.02111 (8)	0.0222 (4)	
H12	0.1184	0.0189	0.0651	0.027*	
C17	0.08711 (10)	−0.1107 (2)	−0.01958 (9)	0.0267 (4)	
H13	0.0547	−0.2015	−0.0033	0.032*	
C18	0.09617 (11)	−0.1094 (2)	−0.08390 (9)	0.0292 (4)	
H14	0.0702	−0.1994	−0.1121	0.035*	
C19	0.14345 (10)	0.0242 (3)	−0.10691 (8)	0.0286 (4)	
H15	0.1495	0.0268	−0.1511	0.034*	
C20	0.18194 (10)	0.1542 (2)	−0.06551 (8)	0.0230 (4)	
H16	0.2143	0.2445	−0.0821	0.028*	
C21	0.28127 (9)	0.4280 (2)	0.01512 (7)	0.0190 (4)	
C22	0.35283 (10)	0.3533 (2)	0.00407 (8)	0.0233 (4)	
H17	0.3672	0.2329	0.0208	0.028*	
C23	0.40343 (10)	0.4484 (3)	−0.03036 (8)	0.0266 (4)	
H18	0.4515	0.3931	−0.0367	0.032*	
C24	0.38387 (10)	0.6248 (3)	−0.05563 (8)	0.0282 (4)	
H19	0.4185	0.6911	−0.0790	0.034*	
C25	0.31309 (10)	0.7026 (3)	−0.04624 (8)	0.0258 (4)	
H20	0.2988	0.8223	−0.0636	0.031*	
C26	0.26302 (10)	0.6055 (2)	−0.01144 (8)	0.0219 (4)	
H21	0.2149	0.6612	−0.0054	0.026*	
C27	0.15982 (10)	0.4430 (2)	0.08339 (8)	0.0195 (4)	
C28	0.18757 (10)	0.5898 (2)	0.12474 (8)	0.0222 (4)	
H22	0.2419	0.6043	0.1354	0.027*	
C29	0.13847 (11)	0.7138 (2)	0.15035 (8)	0.0256 (4)	
H23	0.1594	0.8127	0.1774	0.031*	
C30	0.05859 (11)	0.6942 (3)	0.13665 (8)	0.0277 (4)	
H24	0.0247	0.7785	0.1543	0.033*	
C31	0.02944 (10)	0.5507 (3)	0.09706 (9)	0.0275 (4)	
H25	−0.0250	0.5348	0.0876	0.033*	
C32	0.07955 (10)	0.4284 (2)	0.07081 (8)	0.0241 (4)	
H26	0.0581	0.3313	0.0432	0.029*	
N1	0.26879 (8)	0.1851 (2)	0.10506 (6)	0.0194 (3)	
N2	0.30188 (9)	0.2706 (2)	0.16050 (7)	0.0285 (4)	
N3	0.34303 (8)	−0.02185 (19)	0.15713 (6)	0.0204 (3)	
N4	0.52992 (8)	−0.6447 (2)	0.23468 (7)	0.0238 (3)	
N5	0.53680 (8)	−0.7720 (2)	0.19453 (7)	0.0245 (3)	

### Atomic displacement parameters (Å^2^)

**Table:**

	*U*^11^	*U*^22^	*U*^33^	*U*^12^	*U*^13^	*U*^23^
B1	0.0204 (9)	0.0185 (9)	0.0176 (9)	0.0035 (8)	0.0006 (7)	0.0034 (8)
C1	0.0226 (8)	0.0201 (8)	0.0159 (8)	0.0007 (8)	0.0001 (6)	0.0013 (7)
C2	0.0378 (10)	0.0253 (10)	0.0205 (9)	0.0030 (9)	−0.0045 (8)	−0.0015 (7)
C3	0.0211 (8)	0.0176 (8)	0.0210 (8)	0.0019 (7)	0.0000 (7)	0.0034 (7)
C4	0.0351 (10)	0.0244 (9)	0.0166 (8)	0.0058 (8)	0.0005 (7)	−0.0011 (7)
C5	0.0369 (10)	0.0268 (10)	0.0165 (9)	0.0035 (8)	−0.0036 (7)	0.0015 (7)
C6	0.0216 (8)	0.0229 (9)	0.0190 (8)	0.0015 (8)	−0.0002 (7)	0.0034 (7)
C7	0.0240 (8)	0.0227 (9)	0.0189 (8)	0.0028 (8)	0.0030 (7)	−0.0001 (7)
C8	0.0227 (8)	0.0266 (9)	0.0151 (8)	0.0009 (8)	−0.0005 (6)	0.0020 (7)
C9	0.0232 (9)	0.0229 (9)	0.0222 (9)	0.0031 (8)	0.0057 (7)	0.0046 (7)
C10	0.0266 (9)	0.0245 (9)	0.0235 (9)	0.0028 (8)	0.0050 (7)	0.0029 (7)
C11	0.0296 (10)	0.0304 (10)	0.0285 (10)	0.0027 (9)	0.0023 (8)	0.0071 (8)
C12	0.0319 (10)	0.0275 (10)	0.0409 (12)	0.0094 (9)	0.0102 (9)	0.0093 (9)
C13	0.0449 (12)	0.0287 (10)	0.0347 (11)	0.0094 (10)	0.0140 (9)	0.0002 (9)
C14	0.0403 (11)	0.0302 (10)	0.0234 (9)	0.0054 (9)	0.0041 (8)	0.0009 (8)
C15	0.0192 (8)	0.0190 (8)	0.0196 (8)	0.0061 (7)	0.0001 (6)	0.0014 (7)
C16	0.0214 (9)	0.0234 (9)	0.0215 (9)	0.0041 (8)	0.0016 (7)	0.0018 (7)
C17	0.0224 (9)	0.0216 (9)	0.0343 (10)	0.0007 (8)	−0.0031 (7)	0.0026 (8)
C18	0.0296 (10)	0.0219 (9)	0.0325 (10)	0.0030 (8)	−0.0102 (8)	−0.0068 (8)
C19	0.0350 (10)	0.0300 (10)	0.0190 (9)	0.0061 (9)	−0.0031 (7)	−0.0029 (7)
C20	0.0263 (9)	0.0220 (9)	0.0203 (9)	0.0025 (8)	0.0009 (7)	0.0001 (7)
C21	0.0223 (8)	0.0209 (8)	0.0129 (8)	−0.0009 (7)	−0.0008 (6)	−0.0022 (6)
C22	0.0241 (9)	0.0234 (9)	0.0225 (9)	0.0027 (8)	0.0029 (7)	0.0009 (7)
C23	0.0237 (9)	0.0305 (10)	0.0266 (9)	0.0036 (8)	0.0071 (7)	0.0002 (8)
C24	0.0284 (9)	0.0334 (10)	0.0238 (9)	−0.0030 (9)	0.0081 (7)	0.0029 (8)
C25	0.0303 (9)	0.0259 (9)	0.0205 (9)	0.0009 (8)	0.0008 (7)	0.0047 (7)
C26	0.0224 (8)	0.0251 (9)	0.0181 (8)	0.0030 (8)	0.0017 (7)	−0.0004 (7)
C27	0.0250 (9)	0.0181 (8)	0.0158 (8)	0.0023 (8)	0.0041 (6)	0.0037 (7)
C28	0.0248 (9)	0.0217 (9)	0.0205 (9)	0.0030 (8)	0.0044 (7)	0.0025 (7)
C29	0.0379 (10)	0.0204 (9)	0.0194 (9)	−0.0003 (8)	0.0076 (7)	−0.0004 (7)
C30	0.0345 (10)	0.0270 (9)	0.0238 (9)	0.0088 (9)	0.0125 (8)	0.0012 (8)
C31	0.0239 (9)	0.0298 (10)	0.0295 (10)	0.0021 (8)	0.0059 (7)	0.0024 (8)
C32	0.0260 (9)	0.0240 (9)	0.0225 (9)	0.0014 (8)	0.0032 (7)	−0.0008 (7)
N1	0.0217 (7)	0.0208 (7)	0.0153 (7)	−0.0017 (6)	0.0006 (5)	−0.0010 (6)
N2	0.0382 (9)	0.0267 (8)	0.0182 (7)	0.0030 (7)	−0.0063 (6)	−0.0037 (6)
N3	0.0231 (7)	0.0211 (7)	0.0165 (7)	0.0009 (6)	0.0003 (6)	0.0000 (6)
N4	0.0265 (8)	0.0247 (8)	0.0201 (7)	0.0031 (7)	0.0015 (6)	0.0025 (6)
N5	0.0269 (8)	0.0261 (8)	0.0203 (7)	0.0038 (7)	0.0023 (6)	0.0023 (6)

### Geometric parameters (Å, °)

**Table:**

B1—C15	1.621 (2)	C15—C20	1.401 (2)
B1—C21	1.623 (2)	C16—C17	1.387 (2)
B1—C27	1.631 (2)	C16—H12	0.9500
B1—N1	1.636 (2)	C17—C18	1.383 (3)
C1—N1	1.312 (2)	C17—H13	0.9500
C1—N3	1.349 (2)	C18—C19	1.385 (3)
C1—H1	0.9500	C18—H14	0.9500
C2—N2	1.302 (2)	C19—C20	1.387 (2)
C2—N3	1.363 (2)	C19—H15	0.9500
C2—H2	0.9500	C20—H16	0.9500
C3—C4	1.388 (2)	C21—C22	1.400 (2)
C3—C8	1.385 (2)	C21—C26	1.404 (2)
C3—N3	1.444 (2)	C22—H17	0.9500
C4—C5	1.383 (2)	C22—C23	1.385 (2)
C4—H3	0.9500	C23—C24	1.391 (2)
C5—C6	1.393 (2)	C23—H18	0.9500
C5—H4	0.9500	C24—C25	1.387 (2)
C6—C7	1.394 (2)	C24—H19	0.9500
C6—N4	1.428 (2)	C25—C26	1.390 (2)
C7—H5	0.9500	C25—H20	0.9500
C7—C8	1.381 (2)	C26—H21	0.9500
C8—H6	0.9500	C27—C28	1.408 (2)
C9—C10	1.392 (2)	C27—C32	1.393 (2)
C9—C14	1.393 (2)	C28—C29	1.384 (2)
C9—N5	1.433 (2)	C28—H22	0.9500
C10—C11	1.380 (2)	C29—C30	1.392 (3)
C10—H7	0.9500	C29—H23	0.9500
C11—C12	1.393 (3)	C30—C31	1.377 (3)
C11—H8	0.9500	C30—H24	0.9500
C12—C13	1.383 (3)	C31—C32	1.394 (2)
C12—H9	0.9500	C31—H25	0.9500
C13—C14	1.384 (3)	C32—H26	0.9500
C13—H10	0.9500	N1—N2	1.3787 (18)
C14—H11	0.9500	N4—N5	1.2563 (19)
C15—C16	1.398 (2)		
			
B1—C15—C16	121.02 (14)	C15—C20—C19	122.11 (17)
B1—C15—C20	122.99 (15)	C15—C20—H16	118.9
B1—C21—C22	122.41 (15)	C16—C15—C20	115.98 (15)
B1—C21—C26	121.19 (14)	C16—C17—C18	119.75 (17)
B1—C27—C32	122.56 (15)	C16—C17—H13	120.1
B1—C27—C28	121.70 (15)	C17—C16—H12	118.7
B1—N1—C1	129.79 (13)	C17—C18—C19	119.48 (16)
B1—N1—N2	120.24 (13)	C17—C18—H14	120.3
C1—N1—N2	109.25 (13)	C18—C17—H13	120.1
C1—N3—C2	104.76 (14)	C18—C19—C20	120.08 (17)
C1—N3—C3	129.28 (14)	C18—C19—H15	120.0
C2—N2—N1	104.99 (14)	C19—C18—H14	120.3
C2—N3—C3	125.84 (14)	C19—C20—H16	118.9
C3—C4—C5	118.81 (16)	C20—C19—H15	120.0
C3—C4—H3	120.6	C21—B1—C27	112.21 (14)
C3—C8—C7	119.23 (15)	C21—B1—N1	105.60 (12)
C3—C8—H6	120.4	C21—C22—C23	122.48 (16)
C4—C3—C8	121.50 (16)	C21—C22—H17	118.8
C4—C3—N3	118.53 (15)	C21—C26—C25	122.20 (16)
C4—C5—C6	120.56 (16)	C21—C26—H21	118.9
C4—C5—H4	119.7	C22—C21—C26	116.02 (15)
C5—C4—H3	120.6	C22—C23—C24	120.08 (16)
C5—C6—C7	119.60 (16)	C22—C23—H18	120.0
C5—C6—N4	115.79 (14)	C23—C22—H17	118.8
C6—C5—H4	119.7	C23—C24—C25	119.10 (16)
C6—C7—C8	120.25 (16)	C23—C24—H19	120.4
C6—C7—H5	119.9	C24—C23—H18	120.0
C6—N4—N5	114.42 (14)	C24—C25—C26	120.12 (16)
C7—C6—N4	124.60 (15)	C24—C25—H20	119.9
C7—C8—H6	120.4	C25—C24—H19	120.4
C8—C3—N3	119.91 (14)	C25—C26—H21	118.9
C8—C7—H5	119.9	C26—C25—H20	119.9
C9—C10—C11	119.59 (17)	C27—B1—N1	107.85 (13)
C9—C10—H7	120.2	C27—C28—C29	122.30 (16)
C9—C14—C13	119.91 (17)	C27—C28—H22	118.9
C9—C14—H11	120.0	C27—C32—C31	122.65 (17)
C9—N5—N4	113.92 (14)	C27—C32—H26	118.7
C10—C9—C14	120.07 (16)	C28—C27—C32	115.62 (15)
C10—C9—N5	124.35 (16)	C28—C29—C30	120.25 (17)
C10—C11—C12	120.43 (18)	C28—C29—H23	119.9
C10—C11—H8	119.8	C29—C28—H22	118.9
C11—C10—H7	120.2	C29—C30—C31	119.00 (16)
C11—C12—C13	119.84 (18)	C29—C30—H24	120.5
C11—C12—H9	120.1	C30—C29—H23	119.9
C12—C11—H8	119.8	C30—C31—C32	120.17 (17)
C12—C13—C14	120.13 (18)	C30—C31—H25	119.9
C12—C13—H10	119.9	C31—C30—H24	120.5
C13—C12—H9	120.1	C31—C32—H26	118.7
C13—C14—H11	120.0	C32—C31—H25	119.9
C14—C9—N5	115.58 (15)	N1—C1—N3	109.09 (14)
C14—C13—H10	119.9	N1—C1—H1	125.5
C15—B1—C21	110.99 (13)	N2—C2—N3	111.91 (15)
C15—B1—C27	113.89 (14)	N2—C2—H2	124.0
C15—B1—N1	105.67 (13)	N3—C1—H1	125.5
C15—C16—C17	122.59 (16)	N3—C2—H2	124.0
C15—C16—H12	118.7		
			
B1—C15—C16—C17	179.90 (15)	C20—C15—B1—C21	−9.8 (2)
B1—C15—C20—C19	−179.44 (15)	C20—C15—B1—C27	118.01 (17)
B1—C21—C22—C23	173.63 (15)	C20—C15—B1—N1	−123.80 (16)
B1—C21—C26—C25	−173.56 (15)	C20—C15—C16—C17	0.9 (2)
B1—C27—C32—C31	−176.03 (16)	C20—C19—C18—C17	0.6 (3)
B1—N1—C1—N3	170.60 (14)	C22—C21—B1—C15	−75.75 (19)
B1—N1—N2—C2	−171.42 (15)	C22—C21—B1—C27	155.54 (15)
C1—N1—B1—C15	22.6 (2)	C22—C21—B1—N1	38.3 (2)
C1—N1—B1—C21	−95.12 (19)	C22—C21—C26—C25	−0.5 (2)
C1—N1—B1—C27	144.72 (16)	C22—C23—C24—C25	−0.5 (3)
C1—N1—N2—C2	−0.18 (19)	C24—C23—C22—C21	−0.2 (3)
C1—N3—C2—N2	0.4 (2)	C24—C25—C26—C21	−0.2 (3)
C1—N3—C3—C4	−156.01 (17)	C26—C21—B1—C15	96.88 (17)
C1—N3—C3—C8	26.6 (3)	C26—C21—B1—C27	−31.8 (2)
C2—N3—C1—N1	−0.54 (18)	C26—C21—B1—N1	−149.07 (14)
C2—N3—C3—C4	28.5 (3)	C26—C21—C22—C23	0.6 (2)
C2—N3—C3—C8	−148.83 (17)	C26—C25—C24—C23	0.6 (3)
C3—C4—C5—C6	−1.8 (3)	C27—C28—C29—C30	1.3 (3)
C3—C8—C7—C6	−2.0 (3)	C28—C27—B1—C15	−176.33 (14)
C3—N3—C1—N1	−176.73 (15)	C28—C27—B1—C21	−49.2 (2)
C3—N3—C2—N2	176.80 (15)	C28—C27—B1—N1	66.73 (19)
C5—C6—C7—C8	1.7 (3)	C28—C27—C32—C31	0.1 (2)
C6—N4—N5—C9	178.72 (14)	C29—C28—C27—B1	175.07 (15)
C7—C6—C5—C4	0.2 (3)	C29—C28—C27—C32	−1.1 (2)
C7—C8—C3—C4	0.4 (3)	C30—C31—C32—C27	0.7 (3)
C7—C8—C3—N3	177.64 (15)	C31—C30—C29—C28	−0.4 (3)
C8—C3—C4—C5	1.5 (3)	C32—C27—B1—C15	−0.4 (2)
C10—C9—C14—C13	−1.7 (3)	C32—C27—B1—C21	126.77 (17)
C10—C9—N5—N4	7.0 (2)	C32—C27—B1—N1	−117.34 (16)
C10—C11—C12—C13	−1.0 (3)	C32—C31—C30—C29	−0.6 (3)
C12—C11—C10—C9	0.1 (3)	N1—N2—C2—N3	−0.2 (2)
C12—C13—C14—C9	0.8 (3)	N2—N1—B1—C15	−168.22 (13)
C14—C9—C10—C11	1.3 (3)	N2—N1—B1—C21	74.09 (17)
C14—C9—N5—N4	−173.24 (16)	N2—N1—B1—C27	−46.06 (19)
C14—C13—C12—C11	0.6 (3)	N2—N1—C1—N3	0.46 (18)
C15—C20—C19—C18	−0.3 (3)	N3—C3—C4—C5	−175.80 (16)
C16—C15—B1—C21	171.30 (14)	N4—C6—C5—C4	178.77 (16)
C16—C15—B1—C27	−60.91 (19)	N4—C6—C7—C8	−176.73 (15)
C16—C15—B1—N1	57.29 (18)	N5—C9—C10—C11	−178.98 (16)
C16—C15—C20—C19	−0.5 (2)	N5—C9—C14—C13	178.52 (17)
C16—C17—C18—C19	−0.2 (3)	N5—N4—C6—C5	166.55 (16)
C18—C17—C16—C15	−0.6 (2)	N5—N4—C6—C7	−15.0 (2)

### Hydrogen-bond geometry (Å, °)

**Table:**

*D*—H···*A*	*D*—H	H···*A*	*D*···*A*	*D*—H···*A*
C4—H3···Cg1^i^	0.95	2.91	3.822 (2)	162

Symmetry codes: (i) −*x*+1/2, *y*−1/2, −*z*+1/2.

## References

[bb1] Higashi, T. (2001). *ABSCOR* Rigaku Corporation, Tokyo, Japan.

[bb2] Rasmussen, P. H., Ramanujam, P. S., Hvilsted, S. & Berg, R. H. (1999). *J. Am. Chem. Soc.* **121**, 4738–4743.

[bb3] Rigaku (1998). *PROCESS-AUTO* Rigaku Corporation,Tokyo, Japan.

[bb4] Rigaku/MSC (2002). *CrystalClear* Rigaku/MSC, The Woodlands, Texas, USA, and Rigaku Corporation, Tokyo, Japan.

[bb5] Sheldrick, G. M. (2008). *Acta Cryst.* A**64**, 112–122. 10.1107/S0108767307043930 18156677

[bb6] Wakita, K. (2000). *Yadokari-XG* Department of Chemistry, Graduate School of Science, University of Tokyo, Japan.

